# Brain metastases free survival differs between breast cancer subtypes

**DOI:** 10.1038/bjc.2011.597

**Published:** 2012-01-10

**Authors:** A Berghoff, Z Bago-Horvath, C De Vries, P Dubsky, U Pluschnig, M Rudas, A Rottenfusser, M Knauer, H Eiter, F Fitzal, K Dieckmann, R M Mader, M Gnant, C C Zielinski, G G Steger, M Preusser, R Bartsch

**Affiliations:** 1 Comprehensive Cancer Center Vienna, Medical University of Vienna, Waehringer Guertel 18-20, A-1090 Vienna, Austria; 2 Department of Medicine I, Clinical Division of Oncology, Medical University of Vienna, Waehringer Guertel 18-20, A-1090 Vienna, Austria; 3 Department of Pathology, Medical University of Vienna, Vienna, Austria; 4 Department of Surgery, Medical University of Vienna, Vienna, Austria; 5 Department of Radiotherapy, Medical University of Vienna, Vienna, Austria; 6 Department of Surgery, Academic Teaching Hospital Feldkirch, Feldkirch, Austria; 7 Department of Radiotherapy, Academic Teaching Hospital Feldkirch, Feldkirch, Austria

**Keywords:** advanced breast cancer, brain metastases, carcinomatous meningitis, human epidermal growth factor receptor 2 (HER-2)-positive breast cancer, triple-negative disease

## Abstract

**Background:**

Brain metastases (BM) are frequently diagnosed in patients with HER-2-positive metastatic breast cancer; in addition, an increasing incidence was reported for triple-negative tumours. We aimed to compare brain metastases free survival (BMFS) of breast cancer subtypes in patients treated between 1996 until 2010.

**Methods:**

Brain metastases free survival was measured as the interval from diagnosis of extracranial breast cancer metastases until diagnosis of BM. HER-2 status was analysed by immunohistochemistry and reanalysed by fluorescent *in situ* hybridisation if a score of 2+ was gained. Oestrogen-receptor (ER) and progesterone-receptor (PgR) status was analysed by immunohistochemistry. Brain metastases free survival curves were estimated with the Kaplan–Meier method and compared with the log-rank test.

**Results:**

Data of 213 patients (46 luminal/124 HER-2/43 triple-negative subtype) with BM from breast cancer were available for the analysis. Brain metastases free survival differed significantly between breast cancer subtypes. Median BMFS in triple-negative tumours was 14 months (95% CI: 11.34–16.66) compared with 18 months (95% CI: 14.46–21.54) in HER-2-positive tumours (*P*=0.001) and 34 months (95% CI: 23.71–44.29) in luminal tumours (*P*=0.001), respectively. In HER-2-positive patients, co-positivity for ER and HER-2 prolonged BMFS (26 *vs* 15 m; *P*=0.033); in luminal tumours, co-expression of ER and PgR was not significantly associated with BMFS. Brain metastases free survival in patients with lung metastases was significantly shorter (17 *vs* 21 months; *P*=0.014).

**Conclusion:**

Brain metastases free survival in triple-negative breast cancer, as well as in HER-2-positive/ER-negative, is significantly shorter compared with HER-2/ER co-positive or luminal tumours, mirroring the aggressiveness of these breast cancer subtypes.

In the last decade, overall survival of metastatic breast cancer patients has improved due to advances in systemic treatment (Lin and Winer, 2007; Kiely *et al*, 2011). Despite this success, the rising incidence of brain metastases (BM) as late complication became a major clinical problem (Weil *et al*, 2005; Pestalozzi *et al*, 2006). About 10–15% of all metastatic breast cancer patients will eventually develop symptomatic BM during their course of disease. Brain metastases decrease quality of life and increase morbidity and mortality. Currently, survival of patients with BM ranges from 2 to 16 months (Weil *et al*, 2005).

Prognosis and clinical behaviour of breast cancer differs between subtypes (Perou *et al*, 2000; Sorlie *et al*, 2001; Kennecke *et al*, 2010). Patients with triple-negative tumours, defined by the absence of oestrogen-receptor (ER), progesterone-receptor (PgR) and Her-2-receptor expression, are at higher risk of being diagnosed with BM compared with the luminal or HER-2-positive subtypes (Heitz *et al*, 2009). HER-2-positive patients, on the other hand, have a higher incidence of BM than patients with HER-2-negative breast cancer (Sanna *et al*, 2007). Especially since the introduction of trastuzumab, a growing incidence of symptomatic BM was reported. As trastuzumab cannot penetrate trough the blood–brain barrier due to its molecular weight, a tumour cell sanctuary is created. Furthermore, trastuzumab improves systemic disease control, which leads to a ‘unmasking’ of BM in patients who would otherwise have died from progression of systemic disease.

Apart from triple-negative or HER-2-positive disease, established risk factors for the development of BM are young age at first diagnosis, presence of lung metastases and short disease-free interval (Weil *et al*, 2005).

Treatment of BM remains challenging and consists of surgery, whole-brain irradiation, radiosurgery and systemic therapy (Weil *et al*, 2005). Surgery or radiosurgery is an option for patients with one to three metastases. Whole-brain irradiation, while offering activity also in patients with >3 metastases, causes long-term sides effects such as memory loss and cognitive impairment. Effect of systemic therapy is limited by the blood–brain barrier. Thus, limited therapy options for symptomatic BM substantiates the urgent need for better understanding of risk factors and possibilities of prevention.

Importantly, treatment with lapatinib resulted in a decreased incidence of BM in HER-2-positive disease (Geyer *et al*, 2006). Other preventive measures such as prophylactic cranial radiotherapy, while well established in small-cell-lung cancer, is not routinely used in breast cancer, as no survival benefit was observed so far (Saip *et al*, 2009). Even screening for BM is not a part of routine follow-up, as no evidence for a benefit from early detection exists (Niwinska *et al*, 2007). This, however, might be rather due to the lack of appropriate selection criteria for a potential screening cohort. Therefore, a more precise definition of patients and breast cancer subtypes at high risk for early development of BM is needed (Heitz *et al*, 2009).

The objective of this study therefore was to determine clinical and histopathological risk factors associated with early development of BM. This might identify a high-risk population deriving the largest benefit from screening and prevention.

## Patients and methods

Two Austrian centres contributed information relating to demographics, case history and survival. Data were processed at the Medical University of Vienna, Austria. This retrospective analysis was conducted in accordance with the ethical regulations of the Medical University of Vienna and approval by the local ethics committee was obtained.

### Patients

Patients treated for symptomatic BM from breast cancer between 1996 and 2010 were identified from a breast cancer database. No routine screening for BM was conducted, and none of the patients available for this analysis participated in trials of BM screening or prevention. Data were analysed as of August 2011.

### Hormone-receptor and HER-2 status

Oestrogen-receptor and progesterone-receptor status was assessed by immunohistochemistry (ER*α* antibody, clone 1D5, Dako A/S, Glostrup, Denmark; and PR antibody, Dako A/S). Receptor expression was estimated as the percentage of positively stained tumour cells. Results were given as 1+, 2+ and 3+ positive or negative staining, with a cutoff value of <10% positive tumour cells (Hammond *et al*, 2010). HER-2 status was assessed by immunohistochemistry (Herceptest; Dako A/S) or dual colour fluorescent *in situ* hybridisation (FISH; PathVision HER-2 DNA probe kit, Vysis Inc., Downers Grove, IL, USA). Tumours were classified as HER-2-positive if they had a staining intensity of 3+ on the Herceptest; if a score of 2+ was gained, tumours were reanalysed by FISH (Wolff *et al*, 2007).

### Breast cancer subtypes

Breast cancer subtypes were defined according to the results of the immunohistochemical analysis. Tumours heralding hormone-receptor expression in the absence of HER-2-receptor overexpression were summarised as belonging to the luminal subtype, without further differentiation. The HER-2 subtype was defined by overexpression of the HER-2 receptor and/or amplification of the HER-2/*neu* gene. Tumours were defined as triple-negative in the absence of ER, PgR as well as HER-2 expression (Anders *et al*, 2011; Duan *et al*, 2011).

### Treatment plan and patient evaluation

In metastatic patients, routine re-evaluation of patients’ tumour status was performed every 3 months with contrast-enhanced CT scans of the chest and the abdomen, with additional work up if indicated. In patients with early breast cancer, follow-up was done according to local protocol. Brain imaging was performed only when symptoms of CNS metastases or carcinomatous meningitis occurred. Brain metastases were diagnosed by CT and/or MRI and histologically confirmed in case neurosurgery was performed. Carcinomatous meningitis was defined as enhancement of the meninges as detected by MRI and/or detection of tumour cells in the cerebrospinal fluid. Metastatic breast cancer and BM were treated according to the current evidence-based standard of care including surgery, radiotherapy, systemic therapy, targeted therapy and endocrine treatment (Beslija *et al*, 2007). Follow-up of BM was conducted every 3 months with either contrast-enhanced cranial CT or MRI scans.

### Study end points

We defined brain metastases free survival (BMFS) as the interval from diagnosis of metastatic disease until the development of BM. Therefore, patients with BM as first site of metastatic disease were excluded from analysis of BMFS. Furthermore, we analysed the association of breast cancer subtypes with brain as first site of disease progression, number of BM, time to development of BM (<24 months *vs* >48 months), and development of carcinomatous meningitis.

### Statistical analysis

Brain metastases free survival was estimated by the Kaplan–Meier product limit method. To test the differences between BMFS curves, the log-rank test was used. For correlation of two parameters, the *χ*^2^-test and the likelihood ratio were used. Two-tailed *P*-values <0.05 were considered to indicate statistical significance. Variables exhibiting significance (*P*<0.05) or near significance (*P*<0.09) at univariate analysis were included into a Cox proportional hazards models.

The association of the following variables with BMFS were investigated using univariate analysis: breast cancer subtype (luminal *vs* triple-negative *vs* Her-2-positive), presence of pulmonary metastases, presence of any visceral metastases, age at primary diagnosis (>65 years; <35 years), grading (grades 1 and 2 *vs* 3), stage at primary diagnosis (localised *vs* metastatic) and time to progression after first diagnosis of early breast cancer (<24 months *vs* >24 months). Correlation analysis was performed for subtype and BM as first site of recurrence, time to progression to the brain (<24 months, >48 months), number of BM (1–3 *vs* >3 BM) and presence of carcinomatous meningitis.

All statistics were calculated using statistical package for the social sciences (SPSS) 17.0 software (SPSS Inc., Chicago, IL, USA).

## Results

### Patient characteristics

Overall, 250 patients with BM from breast cancer were identified from two Austrian centres between 1996 and 2010 (absolute incidence of breast cancer in Austria 1996–2010: 68 661 patients). Thirty-seven patients had to be excluded due to incomplete information about breast cancer subtype (e.g., missing data concerning Her-2 status, hormone-receptor status). Therefore, 213 patients were available for this retrospective analysis.

According to the immunohistochemical analysis of the primary tumour, patients were divided into three groups: luminal subtype, HER-2 subtype and triple-negative subtype. Forty-six patients (21.6%) belonged to the luminal subtype, 124 patients (58.2%) to the HER-2 subtype and 43 patients (20.2%) to the triple-negative subtype. Forty-four patients (20.7%) had BM as first site of metastatic disease and therefore were excluded from the analysis of BMFS. All patients were treated according to the current standard of treatment for breast cancer and metastatic breast cancer, respectively (Beslija *et al*, 2007; Goldhirsch *et al*, 2007). In all, 89.9% were treated with chemotherapy-based regime for metastatic disease before the diagnosis of BM. The remaining 10.1% of patients were treated with either endocrine monotherapy or trastuzumab monotherapy. Patient characteristics are summarised in Table 1.

### Brain metastases free survival

**Figure 1:**
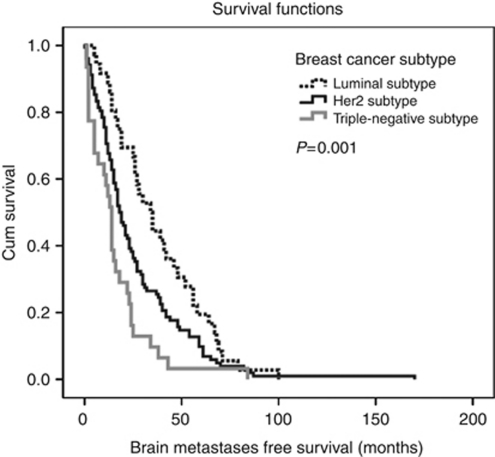
Kaplan–Meier estimates for BMFS. Median BMFS in triple-negative subtype was 14 months (95% CI: 11.34–16.66) compared with 18 months (95% CI: 14.46–21.54) in HER-2 subtype and 34 months (95% CI: 23.71–44.29) in luminal subtype (*P*=0.001, log-rank test).

Median BMFS was 19 months (95% CI: 15.18–22.82) in the population of 169 patients with metastatic breast cancer who did not have BM as first site of progression. Univariate analysis revealed a significant difference in median BMFS between breast cancer subtypes. In the luminal subtype, median BMFS was 34 months (95% CI: 23.71–44.29) compared with 18 months (95% CI: 14.46–21.54) in the HER-2-positive subtype (*P*=0.001, log-rank test) and 14 months (95% CI: 11.34–16.66) in the triple-negative subtype (*P*=0.001, log-rank test) (Figure 1).

**Figure 2:**
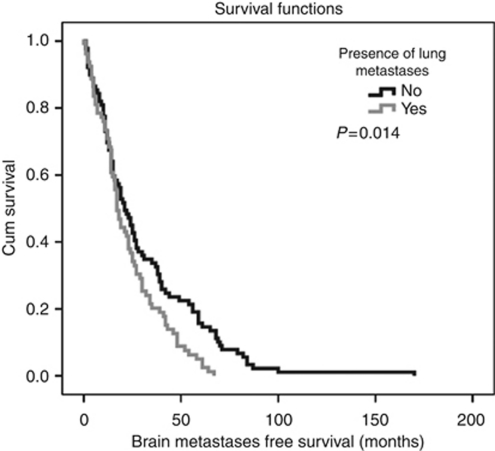
Kaplan–Meier estimates for BMFS. Median BMFS in patients with the presence of lung metastases was 17 months (95% CI: 14.10–19.90) compared with 21 months (95% CI: 15.45–26.55) in patients with no evidence of lung metastases (*P*=0.014, log-rank test).

In patients with lung metastases, median BMFS was 17 months (95% CI: 14.10–19.90) compared with 21 months (95% CI: 15.45–26.55) in patients with no evidence of lung metastases (*P*=0.014, log-rank test) (Figure 2). In patients with time to extracranial progression after first diagnosis of early breast cancer of <24 months, median BMFS was significantly shorter compared to patients with time to extracranial progression after first diagnosis over 24 months (14 *vs* 24 months; *P*<0.001, log-rank test). None of the other variables included into the univariate model displayed a significant influence on BMFS (Table 2).

In the multivariate analysis of BMFS, presence of lung metastases and breast cancer subtype as well as time to extracranial progression after first diagnosis of early breast cancer retained statistical significance. Hazard ratio (HR) for non-luminal breast cancer subtypes was 1.51 (95% CI: 1.17–1.95; *P*=0.002, Cox proportional hazards model), 1.39 (95% CI: 1.01–1.93; *P*=0.047, Cox proportional hazards model) for presence of lung metastases and 1.49 (95% CI: 1.07–2.08; *P*=0.019, Cox proportional hazards model) for time to progression after first diagnosis of early breast cancer of <24 months, respectively.

### *χ*^2^-test and likelihood ratio

The likelihood ratio of developing BM as first site of metastatic disease did not differ significantly between the breast cancer subtypes (luminal subtype 21.7% HER-2 subtype 17.7% triple-negative subtype 20.7% *P*=0.372, *χ*^2^-test).

On the other hand, the likelihood of being diagnosed with BM in <24 months (BMFS <24 months) correlated significantly with the breast cancer subtype. Within the luminal subtype, 30.6% (11 patients) of patients developed BM in <24 months; in the HER-2 subtype, 59.4% (60 patients) and in the triple-negative subtype, 77.4% (24 patients) of patients had a BMFS of <24 months (*P*<0.001, *χ*^2^-test). Furthermore, the likelihood of BMFS >48 months again correlated significantly with the breast cancer subtype. Only one patient (3.2%) within the triple-negative subtype had a BFMS >48 months, while 12 patients (17.6%) of the HER-2 group and 12 patients (33.3%) of the luminal group had a BMFS of >48 months, respectively (*P*=0.006, *χ*^2^-test).

In all, 92 (48.7%) patients had over three BM at first diagnosis of BM. Accordingly, 32.3% of patients had a single metastasis, 9.5% had two BM and 9.5% three BM. The number of BM at time of first diagnosis of BM did not differ between the subtypes. In all, 24 patients (58.5%) within the luminal subtype had three or less metastases, corresponding numbers for the HER-2-positive and triple-negative subtypes are 50.9% and 50.0%, respectively (*P*=0.666, *χ*^2^-test).

The likelihood ratio for the development of carcinomatous meningitis again significantly correlated with breast cancer subtype. In all, 19.6% (nine patients) of the luminal subtype compared with 3.2% (four patients) of the HER-2 subtype and 9.3% (four patients) of the triple-negative subtype developed carcinomatous meningitis (*P*=0.002, *χ*^2^-test).

### BMFS in subsets of the HER-2-positive subtype

In HER-2-positive patients, we further analysed whether HER-2/ER co-positivity or trastuzumab-based therapy had any influence on BMFS. In patients who received trastuzumab-based therapy before the development of BM, median BMFS was 17 months (95% CI: 13.41–20.53) compared with 21 months (95% CI: 8.53–33.47) in HER-2-positive patients who had not received trastuzumab-based treatment (*P*=0.939, log-rank test). Therefore, trastuzumab did not prolong BMFS.

**Figure 3:**
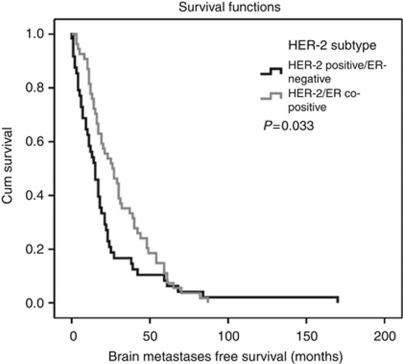
Kaplan–Meier estimates for BMFS. Median BMFS in HER-2/ER co-positive patients was 26 months (95% CI: 16.40–35.60) compared to (15 months; 95% CI: 10.77–19–23) in patients with HER-2-positive/ER-negative disease (*P*=0.033, log-rank test).

In patients with ER/HER-2 co-positive tumours, median BMFS was 26 months (95% CI: 16.40–35.60) and therefore significantly longer than in patients with ER-negative/HER-2-positive disease (15 months; 95% CI: 10.77–19–23; *P*=0.033, log-rank test) (Figure 3).

In a further step, we investigated whether palliative endocrine therapy in ER/HER-2 co-positive patients had a significant impact on BMFS, as tamoxifen has the ability to pass the blood–brain barrier. Indeed, BMFS in patients who received palliative endocrine therapy was 30 months (95% CI: 16.17–43.83) compared with 14 months (95% CI: 10.31–17.68) months in patients with ER/HER-2 co-positive disease who did not receive prior palliative endocrine therapy (*P*=0.004, log-rank test).

### BMFS in subsets of the luminal subtype

Expression of progesterone receptor did not significantly influence BMFS in patients with breast cancer of the luminal subtype. In PgR-positive patients, median BMFS was 35 months (95% CI: 17.39–52.62) compared with 34 months (95% CI: 18.35–49.66) in PgR-negative patients (*P*=0.692, log-rank test).

### Overall survival after diagnosis of BM

Median overall survival after the diagnosis of BM was 5 months (95% CI: 2.64–7. 36) in the luminal group, 7 months (95% CI: 4.31–969) in HER-2-positive group and 5 months (95% CI: 1.83–8.17) in triple-negative breast cancer patients (*P*=0.364, log-rank test). HER-2-positive patients treated with trastuzumab-based therapy after completion of local therapy for BM (surgery, radiotherapy) had a significant longer overall survival after diagnosis of BM (4 *vs* 14 months; 95% CI: 2.40–5.61 *vs* 7.22–20.78; *P*<0.001, log-rank test).

## Discussion

Brain metastases are an increasing issue in modern breast cancer therapy, as up to 15% of patients with stage IV disease will eventually be diagnosed with symptomatic BM (Weil *et al*, 2005). Therefore, development of adequate preventive strategies is urgently required.

In the field of BM prevention in Her-2-positive disease, promising results of lapatinib were reported, a dual tyrosine-kinase inhibitor of EGFR and HER-2 (Cameron *et al*, 2008). Other preventive strategies such as prophylactic cranial irradiation currently have no role in breast cancer treatment, as supporting data are missing (Saip *et al*, 2009). Also, screening for BM is not established, since early detection of BM was not found to influence survival henceforth (Niwinska *et al*, 2010). This, however, might result from the inclusion of patients at relatively low risk for developing BM into the respective clinical trials; therefore, a better definition of risk groups is warranted as first step to establish effective strategies of screening and prevention.

Clinical and translational research redefined breast cancer as a heterogeneous disease, divided into different subtypes defined by divergent gene expression profiles. In daily clinical practice, grading as well as immunohistochemical assessment of hormone-receptor status, Her-2, and Ki-67 are usually used as approximation. Therefore, breast cancer is assigned to the luminal, the HER-2 or the triple-negative phenotype at first diagnosis. This classification influences estimation of prognosis and treatment decisions (Perou *et al*, 2000; Sorlie *et al*, 2001, 2003). In the present study, we show that different breast cancer subtypes associate with time to development of BM. Patients with triple-negative disease had a significantly shorter BMFS (14 months) compared with 34 months in patients with luminal tumours (*P*=0.001). Previously, the triple-negative subtype was identified to have a higher overall risk of developing BM; furthermore, BM are diagnosed relatively early during the course of disease (Pestalozzi *et al*, 2006; Heitz *et al*, 2009). Here, we could demonstrate tremendous differences of BMFS in triple-negative disease in comparison to luminal tumours, as BMFS of luminal subtypes is almost doubled. This finding indicates that triple-negative breast cancer warrants further research of BM-preventive strategies (Pestalozzi, 2009).

A higher incidence of BM was observed in HER-2-positive disease as well. Different authors suggested a connection to trastuzumab, a monoclonal antibody targeting the extracellular domain of HER-2. As trastuzumab cannot penetrate the blood–brain barrier, the CNS becomes a safe haven for tumour cells (Clayton *et al*, 2004). Also, improved control of systemic disease may eventually lead to the ‘unmasking’ of BM (Lin and Winer, 2007). In our analysis, BMFS within the HER-2 subtype was 18 months and was significantly different from the other two subtypes (*P*=0.001). Compared with luminal cancers, shorter BMFS was observed in Her-2-positive disease, while BMFS was longer compared with triple-negative tumours. No influence of trastuzumab-based therapy on BMFS was observed. This finding indicates that biological behaviour rather than systemic treatment defines the risk for early or late development of BM in patients with HER-2-positive breast cancer (Burstein *et al*, 2005; Pestalozzi *et al*, 2006; Lin and Winer, 2007).

Several studies postulated the absence of ER expression as an unfavourable factor for the probability of developing BM (Slimane *et al*, 2004; Weil *et al*, 2005). Therefore, we performed an analysis of BMFS in the HER-2-positive subtype in dependence of ER expression. Patients with ER/HER-2 co-positive disease were shown to have significantly longer BMFS compared with patients with ER-negative/HER-2-positive disease (26 months *vs* 15 months; *P*=0.033). This once again shows that the Her-2-positive phenotype comprises heterogeneous subtypes.

Brain metastases are usually diagnosed rather late in the course of metastatic disease (Weil *et al*, 2005). Previous studies indicate a correlation of visceral and pulmonary metastases and the occurrence of BM (Weil *et al*, 2005; Kennecke *et al*, 2010). Our findings further support this investigation, as pulmonary metastases remained a significant risk factor associated with shorter BMFS in the Cox regression model (HR 1.49; *P*=0.016). Therefore, we suggest that patients with triple-negative tumours and pulmonary metastases might be the most suitable group for prospective trials investigating strategies of screening and prevention.

The number of BM is an important factor for prognosis as well as treatment, as surgery or radiosurgery is usually only applied in patients with oligometastatic (1–3 metastases) disease (Kamar and Posner, 2010; Niwinska *et al*, 2011a, 2011b). Recently, an influence of breast cancer subtypes on the number of BM at first diagnosis was postulated. Oestrogen-receptor-positive patients, according to one study, might be more likely to develop oligometastatic brain involvement (Garg *et al*, 2011). In our homogenous, large collective, however, we cannot support those findings; the likelihood for oligometastatic involvement did not differ between the breast cancer subtypes.

Carcinomatous meningitis, just like BM, occurs late during the course of the disease and treatment options are very limited (de Azevedo *et al*, 2011). While breast cancer subtype influences overall survival after the diagnosis of carcinomatous meningitis, little is known about risk factors (Lee *et al*, 2011; Niwinska *et al*, 2011a, 2011b). In our study, patients with luminal subtype were at higher risk for the development of carcinomatous meningitis compared to patients with HER-2 or triple-negative disease (19.6% *vs* 3.2% *vs* 9.3% *P*=0.002). Although the small sample size has to be taken into account, this apparent contradiction to solid BM warrants further investigation.

In conclusion, our study shows that patients with triple-negative as well as patients with ER-negative/HER-2-positive disease are at highest risk for developing BM early during their course of disease. The risk is further raised by the presence of pulmonary metastases. This analysis might help in defining the optimal breast cancer patient population for future prospective trials of BM screening and prevention.

## Figures and Tables

**Table 1:** Patient characteristics (a) without BM and (b) with BM as first site of progression

**(a)**			
	**Entered patients (*n*=169)**		
**Characteristics**	***n***		**%**
Median age at first diagnosis (years)		50	
Range		25–82	
Age >65 years	17		10.1
Age <35 years	17		10.1
			
Grade 3 tumour	116		73.9
Invasive ductal carcinoma	135		87.1
Stage IV	32		18.9
			
*Subtype*			
Luminal subtype	36		21.3
HER-2 subtype	102		60.4
Triple-negative subtype	31		18.3
			
Adjuvant chemotherapy	115		83.9
Adjuvant endocrine therapy	53		38.1
Adjuvant trastuzumab	12		8.8
Median time to progression (months)		22	
Range		0–166	
Visceral metastases	121		72.0
Brain as the first site of metastatic disease	0		0
			
Median metastatic sites		2	
Range		1–5	
Lung	79		47.0
Liver	71		42.3
Bones	81		48.2
Lymph nodes	48		28.6
Soft tissue	54		32.1
Skin	17		10.1
Others	11		6.6
			
Palliative chemotherapy before BM	152		89.9
Palliative endocrine therapy before BM	63		37.5
Palliative trastuzumab before BM	85		50.3
Palliative lapatinib before BM	2		1.2
			
*Response to systemic therapy at time of BM diagnosis*			
CR	3		3.3
PR	29		31.9
SD	32		35.2
PD	27		29.7
			
Median BM free survival (months)		19	
Range		1–170	
Median OS from first diagnosis (months)		58.5	
Range		3–218	
Median OS from diagnosis of metastatic disease		33	
Range		2–125	
Median OS from diagnosis of BM (months)		5.5	
Range		0–81	
			
**(b)**			
	**Entered patients (*n*=44)**		
**Characteristics**	***n***		**%**
Median age at first diagnosis (years)		54	
Range		27–79	
Age >65 years	5		11.4
Age <35 years	4		9.1
			
Grade 3 tumour	31		75.6
Invasive ductal carcinoma	30		73.2
Stage IV	4		9.1
			
*Subtype*			
Luminal subtype	10		22.7
HER-2 subtype	22		50.0
Triple-negative subtype	12		27.3
Adjuvant chemotherapy	33		80.5
Adjuvant endocrine therapy	13		32.5
Adjuvant trastuzumab	4		10.0
median time to progression (months)		18.5	
Range		0–89	
Visceral metastases	17		38.6
Brain as only site of metastatic disease	22		50
			
Median metastatic sites		1	
Range		1–6	
Lung	6		13.6
Liver	15		34.1
Bones	11		25.0
Lymph nodes	6		13.6
Soft tissue	4		9.1
Skin	0		0
			
Others	1		2.3
Median OS from first diagnosis (months)		29	
Range		0–121	
Median OS from diagnosis of metastatic disease (months)		9	
Range		0–50	
Median OS from diagnosis of BM (months)		9	
Range		0–50	

Abbreviations: CR=complete response; PR=partial response; SD=stable disease; PD=progressive disease; BM=brain metastases; OS=overall survival.

Characteristics grading, staging, subtype are from time point of first diagnosis. Characteristics metastatic sites are from time point of diagnosis of brain metastases.

**Table 2:** Univariate analysis: factors associated with brain metastases free survival (BMFS)

**Factor**	**Median BMFS (months)**	**95% CI**	***P*-value**
*Subtype*			
Luminal subtype	34	23.71–44.29	0.001
HER-2 subtype	18	14.47–21.54	
Triple-negative subtype	14	11.34–16.66	
			
*Presence of metastases*			
Visceral	18	12.97–23.03	n.s.
Pulmonary	17	14.10–19.90	0.014
			
*Age at first diagnosis*			
<35 years	16	11.97–20.03	n.s.
> 65 years	21	8.90–33.10	n.s.
			
Grade 3	17	13.70–20.30	n.s.
Stage IV at primary diagnosis	19	9.02–28.98	n.s.
Time to progression <24 months	14	12.09–15.91	<0.001

Abbreviation: CI=confidence interval.
